# The Reaction of Ethyl 2-oxo-2*H*-chromene-3-carboxylate with Hydrazine Hydrate 

**DOI:** 10.3390/molecules18022084

**Published:** 2013-02-06

**Authors:** Hatem A. Abdel-Aziz, Tilal Elsaman, Mohamed I. Attia, Amer M. Alanazi

**Affiliations:** 1 Department of Pharmaceutical Chemistry, College of Pharmacy, King Saud University, P.O. Box 2457, Riyadh 11451, Saudi Arabia; 2 Department of Applied Organic Chemistry, National Research Center, Dokki, Cairo 12622, Egypt; 3 Department of Medicinal and Pharmaceutical Chemistry, National Research Center, Dokki, Cairo 12622, Egypt

**Keywords:** salicylaldehyde azine, malonohydrazide, coumarins, hydrazides/hydrazones, ring-opening

## Abstract

Although salicylaldehyde azine (**3**) was reported in 1985 as the single product of the reaction of ethyl 2-oxo-2*H*-chromene-3-carboxylate (**1**) with hydrazine hydrate, we identified another main reaction product, besides **3**, which was identified as malono-hydrazide (**4**). In the last two decades, however, some articles have claimed that this reaction afforded exclusively hydrazide **2** and they have reported the use of this hydrazide **2** as a precursor in the syntheses of several heterocyclic compounds and hydrazones **6**. We reported herein a study of the formation of **2** and a facile route for the synthesis of the target compounds *N'*-arylidene-2-oxo-2*H*-chromene-3-carbohydrazides **6a**–**f**.

## 1. Introduction

The hydrazides are very useful starting materials for the construction of several functionalized heterocycles with a broad spectrum of biological activities, and consequently they have been studied in considerable detail over the decades [1,2]. For instance, hydrazides are versatile raw materials to synthesize pyrroles [3], pyrazoles [4], 1,3-thiazoles [5], 1,3,4-oxadiazoles [6], 1,2,4-triazoles [7], 1,3,4-thiadiazoles [8], 1,2,4-triazolo[3,4-*b*]-1,3,4-thiadiazoles [9] and 1,2,4-triazolo[3,4-*b*]-1,3,4-thiadiazines [10]. The common practical route for hydrazide synthesis is the treatment of esters with hydrazine hydrate. The use of microwave irradiation is a recent contribution to this field for the facile preparation of hydrazides by solvent-free reactions of acid derivatives with hydrazine hydrate [11,12]. On the other hand, there is a great deal of interest in chromene-2-ones, especially 3-substituted derivatives, due to their important pharmacological effects, including analgesic, anti-arthritis, anti-inflammatory, anti-pyretic, anti-viral, anti-cancer and anticoagulant properties [13,14,15]. In the light of previous data and in continuation of our interest in the chemistry of hydrazides [16,17,18,19,20,21,22,23,24], our efforts targeted the synthesis of hydrazide **2** through the title reaction according to the literature methods [25,26,27,28,29,30,31,32,33,34,35,36], with the intention of subsequently treating hydrazide **2** with different aldehydes to obtain hydrazones **6a**–**f** for our biological screening program. We report herein the results achieved to this end.

## 2. Results and Discussion

The contradictory data reported in last two decades [25,26,27,28,29,30,31,32,33,34,35,36], alerted us to be careful in our preparation of hydrazide **2**, for instance the hours of reflux used in the title reaction were 2 h [28,33], 3 h [29], 4 h [30], 6 h [27,36] and 10 h [26,34], and afforded different yields of the claimed hydrazide **2**: 99% [25], 90% [26], 80% [27,28,29,30], 72% [32], 65% [31] and 62% [34]. Moreover, different melting points have been reported for the claimed hydrazide **2** (m.p. 136–138 °C [26], 145 °C [32], 206–209 °C [30]).

When we reacted an equimolar ratio of ester **1** with hydrazine hydrate at reflux and followed the reaction progress by TLC, the spot of ester **1** still remained, even after refluxing for 20 h. However, the ester **1** was completely consumed after refluxing 2 h when we used excess hydrazine hydrate (2.5 equiv.). In this case, after cooling, we isolated yellow crystals with melting point 205–206 °C.

The IR, mass and NMR spectral data did not coincide however with those of the expected hydrazide **2**. For example, the ^1^H-NMR of our yellow crystals revealed one D_2_O-exchangeable signal, whereas there were no carbonyl resonances in the ^13^C-NMR data. In addition, its IR spectrum showed no evidence of the characteristic lactone carbonyl absorption band, while the mass spectrum of these crystals showed a peak at *m/z* 240.5 and not the expected one at *m/z* 204 corresponding to the molecular ion of claimed hydrazide **2**.

**Scheme 1:**
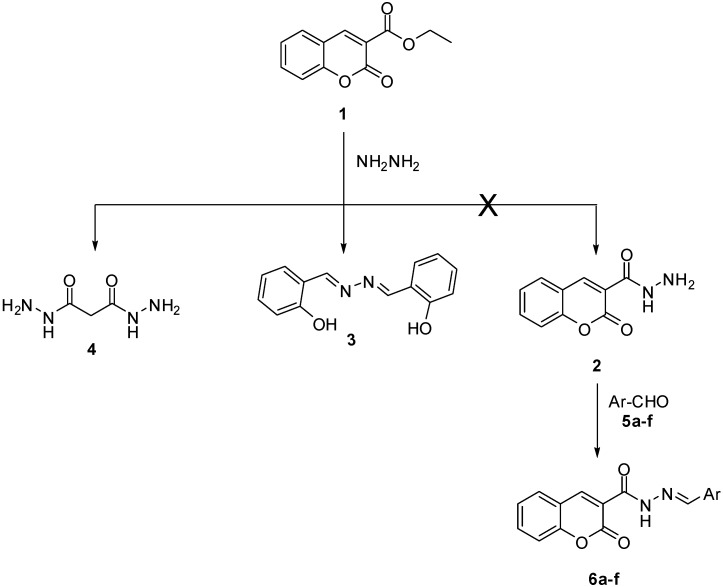
The reaction of ester **1** with hydrazine hydrate.

In an exhaustive literature survey of the reaction product(s) of ester **1** and hydrazine hydrate, bearing in consideration the possible ring-openings of chromene-2-ones [37], Soliman *et al.* [38] described, in 1985, the reaction of ethyl 2-oxo-2*H*-chromene-3-carboxylate (**1**) with hydrazine hydrate in refluxing ethanol, in which they obtained salicylaldehyde azine (**3**) in 31.5% and 42.4% yields for one and two equivalent of hydrazine hydrate, respectively, instead of the claimed hydrazide **2**. The structure of the well-known and commercially available salicylaldehyde azine (**3**), was rigorously proven by independent synthesis using the reaction of salicylaldehyde with hydrazine hydrate [39]. Comparison of the melting point and spectral data for an authentic sample of compound **3** and the yellow crystals produced in our trials, gave us absolute confidence to assign the structure of these crystals to be salicylaldehyde azine (**3**), in complete agreement with the results of Soliman and his co-workers (Scheme 1).

Although the azine **3**, produced by Soliman *et al.* through the reaction of ester **1** with hydrazine hydrate, proved to be identical with the substance we have produced by the same reaction, in the current work, when the filtrate was evaporated under vacuum, after separation of salicylaldehyde azine (**3**), a white powder was produced, which was recrystallized from ethanol to give colorless flakes with mp. 153–154 °C. ^1^H-NMR of the latter showed a singlet at *δ* 3.41 integrating for 2Hs, in addition to two D_2_O-exchangeable signals at *δ* 4.38 and *δ* 9.07 integrating for 2H and 1H, respectively, whereas its ^13^C-NMR spectrum showed only two peaks, the first of a sp^3^ carbon at *δ* 46.5 and the second for two sp^2^ carbons at *δ* 170.7. The ^1^H- or ^13^C-NMR spectra did not show any aromatic protons or carbons, respectively. The mass spectrum of the latter compound exhibited a peak at *m/z* 132.3.

**Scheme 2:**
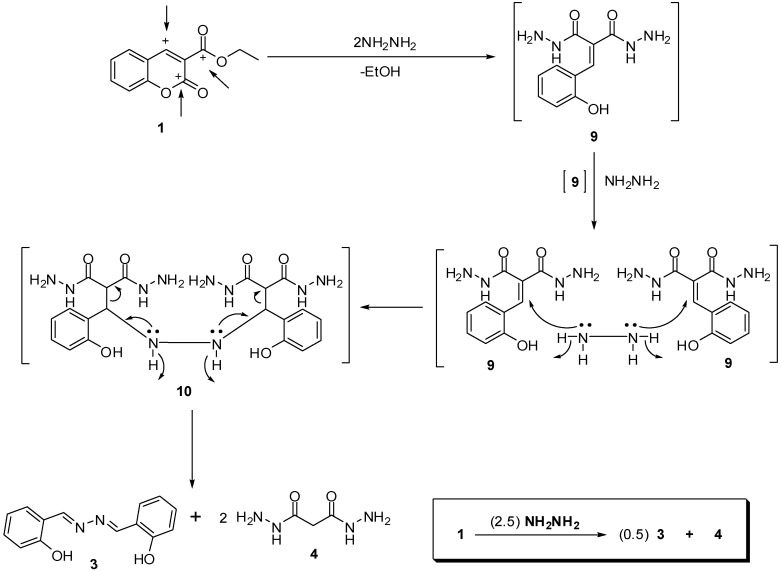
The proposed mechanism for the reaction of ester **1** with hydrazine hydrate and its molar ratio equation.

Furthermore, on the basis of formation of salicylaldehyde azine (**3**) and according to the reported ring-opening behavior of the 2*H*-chromene moiety, we can propose a mechanism for the formation of compound **3** and we can predict the structure of the obtained unknown compound in the title reaction. 2*H*-1-Benzopyran-2-one (coumarin) derivatives are highly reactive because the pyran-2-one ring is an aliphatic moiety that is likely to undergo ring-opening under nucleophilic attack at the lactone acyl centre or nucleophilic conjugate addition at the carbon-carbon double bond [37]. Therefore, in ester **1** we have two nucleophilic centers in addition to the carbonyl function in the ester side chain (Scheme 2).

The previous facts led our proposed mechanism to suppose malonohydrazide (**4**) to be the structure of the compound isolated from the filtrate of the title reaction. In addition, comparing analytical data of the isolated malonohydrazide (**4**) with that of authentic sample of malonohydrazide (m.p. 151–153 °C) [40] revealed them to be identical in all aspects. Moreover, the proposed mechanism proved the needed molar ratio of hydrazine hydrate (2.5 mol equiv.) to achieve full consumption of ester **1**. However, compounds **3** and **4** were also isolated when the reaction proceeded at 0–5 °C.

**Scheme 3:**
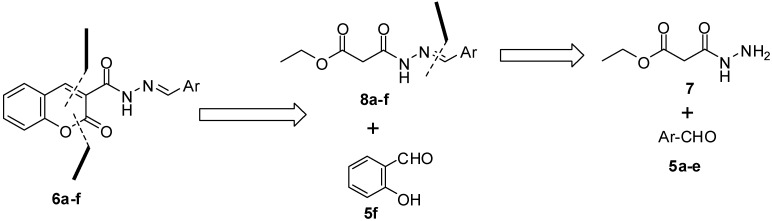
Retrosynthetic analysis of the targeted derivatives **6a**–**f**.

Our attention was next shifted to the synthesis of our targeted hydrazones **6a**–**f** (Scheme 1). Scheme 3 shows a representative retrosynthetic approach for this class of compounds. The ethyl 3-(2-arylidenehydrazinyl)-3-oxopropanoates **8a**–**f** were condensed with salicylaldehyde (**5f**) to generate the targeted hydrazones **6a**–**f**. Compounds **8a**–**f** were synthesized from the reaction of ethyl 3-hydrazinyl-3-oxopropanoate (**7**) with the appropriate aldehydes **5a**–**e**.

**Scheme 4:**
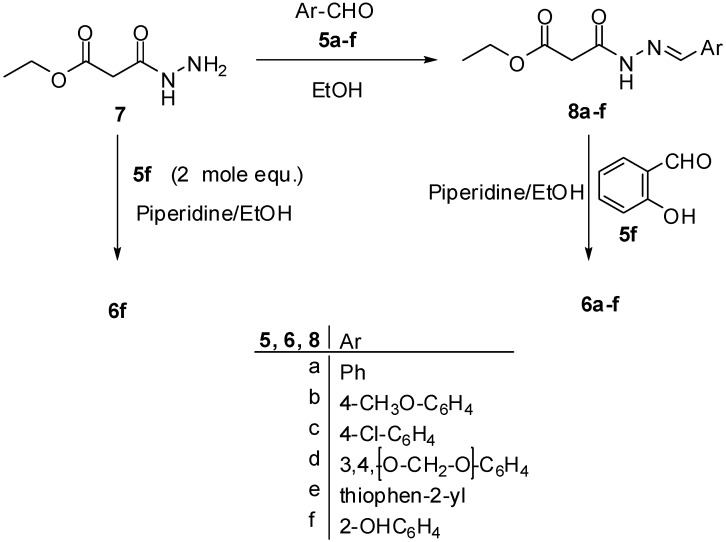
Synthetic route for synthesis of hydrazones **6a**–**f**.

Thus, treatment of ethyl 3-hydrazinyl-3-oxopropanoate (**7**) [38] with the appropriate aldehydes **5a**–**f** in refluxing ethanol yielded the corresponding ethyl 3-(2-arylidenehydrazinyl)-3-oxopropanoates **8a**–**f** (Scheme 4). 

Next, the condensation reaction of hydrazones **8a**–**f** with salicyaldehyde (**5f**) in the presence of piperidine afforded exclusively compounds **6a**–**f**. IR spectra of the latter products revealed two carbonyl absorption bands in the 1704–1699 and 1666–1610 cm^−1^ regions, in addition to the absorption band of NH function in the 3291–3195 cm^−1^ region. Their ^1^H-NMR spectra exhibited NH group D_2_O-exchangeable signals in the δ 11.65–11.95 region, in addition to two singlet signals in the δ 8.38–8.71 and δ 8.90–8.94 regions corresponding to the –CH=C– group of the hydrazone function and pyran ring, respectively. In our hands, the melting points of **8a**, **8b** and **8c** were recorded as 258–260 °C, 265–267 °C and 288–290 °C, respectively, while the reported melting points of the latter claimed compounds 205–207 °C, 194–196 °C and 216–218 °C, respectively [30]. Finally, compound **6f** was synthesized directly by the reaction of ethyl 3-hydrazinyl-3-oxopropanoate (**7**) with two mol equiv. of salicylaldehyde (**5f**) in the presence of piperidine.

## 3. Experimental

### 3.1. General

Infrared (IR) Spectra were recorded as KBr disks using the Perkin Elmer FT-IR Spectrum BX apparatus. Melting points were determined on a Gallenkamp melting point apparatus and are uncorrected. NMR Spectra were scanned in DMSO-*d*_6_ on a Jeol NMR spectrophotometer operating at 400 MHz for ^1^H and 100 MHz for ^13^C. Chemical shifts are expressed in δ-values (ppm) relative to TMS as an internal standard. Coupling constants (*J*) are expressed in Hz. D_2_O was added to confirm the exchangeable protons. Mass spectra were measured on an Agilent Triple Quadrupole 6410 QQQ LC/MS equipped with an ESI (electrospray ionization) source. The elemental analyses were performed at the Microanalytical Center of Cairo University. Ethyl 3-hydrazinyl-3-oxopropanoate (**7**) was prepared according to the reported method (m.p. 68–69 °C) [40].

### 3.2. The Reaction of Ethyl 2-oxo-2H-Chromene-3-carboxylate *(**1**)* with Hydrazine Hydrate

A mixture of ethyl 2-oxo-2*H*-chromene-3-carboxylate (**1**, 2.18 g, 10 mmol) and hydrazine hydrate, (99%, 1.3 g, 25 mmol) in absolute ethanol (50 mL) was heated under reflux for 2 h. The precipitate formed was filtered off, washed with ethanol and dried. Recrystallization from ethanol gave yellow crystalsof *salicyaldehyde azine* (**3**) [38,39] (1.15 g, 48%); m.p. 205–206 °C (204.3 °C) [39]; IR *ν* 3350–2830 (OH), 1633 (C=N) cm^−1^; ^1^H-NMR *δ* 6.84–7.72 (m, 8H, ArH), 8.86 (s, 2H, –CH=), 10.90 (s, D_2_O exch., 2H, OH); ^13^C-NMR *δ* 117.9, 118.7, 121.4, 132.0, 132.5, 159.2, 162.0; MS *m/z*: 240.5 (M^+^). Anal. calcd. for C_14_H_12_N_2_O_2_ (240.26): C, 69.99; H, 5.03; N, 11.66%. Found: C, 70.23; H, 5.17; N, 11.48%. Evaporation of the filtrate of the latter reaction gave a residue, which was washed with 70% ethanol, dried and recrystallized from ethanol to yield colorless flakes of *malonohydrazide* (**4**) [40] (1.14 g, 86%); m.p. 153–154 °C (151–153 °C); IR *ν* 3323–3246 (NH_2_, NH), 1635 (C=O), cm^−1^; ^1^H-NMR *δ* 3.41 (s, 2H, CH_2_), 4.38 (s, D_2_O exch., 4H, 2NH_2_), 9.07 (s, D_2_O exch., 2H, NH); ^13^C-NMR *δ* 46.5 (CH_2_), 170.7 (2C=O); MS *m/z*: 132.3 (M^+^). Anal. calcd. for C_3_H_8_N_4_O_2_ (132.12): C, 27.27; H, 6.10; N, 42.41%. Found: C, 27.04; H, 6.38; N, 42.16%.

### 3.3. General Procedure for the Synthesis of Ethyl 3-(2-arylidenehydrazinyl)-3-oxopropanoates ***8a–f***

A solution of hydrazide **7** (1.46 g, 10 mmol) and the appropriate aldehyde **5a**–**f** (10 mmol) in absolute ethanol (30 mL) was refluxed for 1h and then cooled to 5–10 °C. The solid formed was collected by filtration, washed with ethanol and crystallized from ethanol to afford hydrazones **8a**–**f**.

*Ethyl 3-(2-benzylidenehydrazinyl)-3-oxopropanoate* (**8a**).Yield (2.0 g, 85%); m.p. 115–17 °C (m.p. 111–113 °C [41]); IR *ν* 3292 (NH), 1735 (C=O, ester), 1682 (C=O, amide) cm^−1^; ^1^H-NMR *δ* 1.17 (t, *J* = 6.6 Hz, 3H, CH_3_), 3.65 (s, 2H, CH_2_), 4.09 (q, *J* = 6.6 Hz, 2H, CH_2_), 7.41–7.71 (m, 5H, ArH), 7.96 (s, 1H, –CH=), 11.58 (s, D_2_O exch., 1H, NH); ^13^C-NMR *δ* 14.6 (CH_3_), 41.8 (CH_2_), 61.0 (CH_2_), 127,3, 129.2, 129,3, 129.8, 134.6 (ArC), 143.7 (–CH=), 168.3 (C=O), 168.5 (C=O); MS *m/z*: 257.1 (M^+^+23). Anal. calcd. for C_12_H_14_N_2_O_3_ (234.25): C, 61.53; H, 6.02; N, 11.96%. Found: C, 61.27; H, 5.85; N, 12.19%.

*Ethyl 3-(2-(4-methoxybenzylidene)hydrazinyl)-3-oxopropanoate* (**8b**). Yield (2.46 g, 93%); m.p. 116–118 °C (m.p. 110–111 °C [41]); IR *ν* 3293 (NH), 1728 (C=O, ester), 1681 (C=O, amide) cm^−1^; ^1^H-NMR *δ* 1.16 (t, *J* = 6.6 Hz, 3H, CH_3_), 3.62 (s, 2H, CH_2_), 3.80 (s, 3H, OCH_3_), 4.08 (s, 2H, CH_2_), 4.07 (q, *J* = 6.6, Hz, 2H, CH_2_), 6.98 (d, *J* = 8.8, Hz, 2H, ArH), 7.57 (d, *J* = 8.8 Hz, 2H, ArH), 7.90 (s, 1H, –CH=), 11.44 (s, D_2_O exch., 1H, NH); ^13^C-NMR *δ* 14.6 (CH_3_), 41.7 (CH_2_), 55.9 (OCH_3_), 60.9 (CH_2_), 114.8, 127.2, 128.9, (ArC), 143.5 (–CH=), 161.2 (ArC), 168.2 (C=O), 168.3 (C=O); MS *m/z*: 265.2 (M^+^+1), 287.2 (M^+^+23). Anal. calcd. for C_13_H_16_N_2_O_4_ (264.28): C, 59.08; H, 6.10; N, 10.60%. Found: C, 60.24; H, 6.36; N, 10.57%.

*Ethyl 3-(2-(4-chlorobenzylidene)hydrazinyl)-3-oxopropanoate* (**8c**). Yield (2.23 g, 83%); m.p. 168–170 °C (m.p. 160–163 °C [41]); IR *ν* 3292 (NH), 1733 (C=O, ester), 1675 (C=O, amide) cm^−1^; ^1^H-NMR *δ* 1.16 (t, *J* = 6.6 Hz, 3H, CH_3_), 3.65 (s, 2H, CH_2_), 4.08 (q, *J* = 6.6 Hz, 2H, CH_2_), 7.49 (d, *J* = 8.8 Hz, 2H, ArH), 7.65 (d, *J* = 8.1, Hz, 2H, ArH), 7.96 (s, 1H, –CH=), 11.64 (s, D_2_O exch., 1H, NH); ^13^C-NMR *δ* 14.6 (CH_3_), 41.5 (CH_2_), 61.1 (CH_2_), 128.9, 129.4, 133.6, 134.9 (ArC), 142.4 (–CH=), 168.2 (C=O), 168.5 (C=O); MS *m/z*: 291.1 (M^+^+23). Anal. calcd. for C_12_H_13_ClN_2_O_3_ (268.70): C, 53.64; H, 4.88; N, 10.43%. Found: C, 53.75; H, 5.04; N, 10.25%.

*Ethyl 3-(2-(benzo[d][1,3]dioxol-5-ylmethylene)hydrazinyl)-3-oxopropanoate* (**8d**). Yield (2.37 g, 85%); m.p. 158–160 °C; IR *ν* 3187 (NH), 1736 (C=O, ester), 1676 (C=O, amide) cm^−1^; ^1^H-NMR *δ* 1.17 (t, *J* = 7.36 Hz, 3H, CH_3_), 3.61 (s, 2H, CH_2_), 4.07 (q, *J* = 7.36 Hz, 2H, CH_2_), 6.07 (s, 2H, CH_2_), 6.95 (d, *J* = 8.1 Hz, 1H, ArH), 7.06 (d, *J* = 8.8 Hz, 1H, ArH), 7.22 (s, 1H, ArH), 7.86 (s, 1H, –CH=), 11.46 (s, D_2_O exch., 1H, NH); ^13^C-NMR *δ* 14.6 (CH_3_), 41.8 (CH_2_), 60.9 (CH_2_), 102.1 (CH_2_), 105.2, 108.9, 123.7, 129.1 (ArC), 143.3 (–CH=), 148.5, 149.5 (ArC), 168.30 (C=O), 168.33 (C=O); MS *m/z*: 279.1 (M^+^+1), 301.1 (M^+^+23). Anal. calcd. for C_13_H_14_N_2_O_5_ (278.26): C, 56.11; H, 5.07; N, 10.07%. Found: C, 56.19; H, 4.93; N, 9.86%.

*Ethyl 3-oxo-3-(2-(thiophen-2-ylmethylene)hydrazinyl)propanoate* (**8e**). Yield (1.87 g, 78%); m.p. 118–120 °C; IR *ν* 3292 (NH), 1732 (C=O, ester), 1677 (C=O, amide) cm^−1^; ^1^H-NMR *δ* 1.19 (t, *J* = 7.32 Hz, 3H, CH_3_), 3.57 (s, 2H, CH_2_), 4.09 (q, *J* = 7.32 Hz, 2H, CH_2_), 8.14 (s, 1H, –CH=), 7.10 (t, *J* = 4.4 Hz, 1H, thiophene H), 7.40 (d, *J* = 2.92 Hz, 1H, thiophene H), 7.62 (d, *J* = 4.4 Hz, 1H, thiophene H); 11.56 (s, D_2_O exch., 1H, NH); MS *m/z*: 241.1 (M^+^+1), 263.1 (M^+^+23). Anal. calcd. for C_10_H_12_N_2_O_3_S (240.28): C, 49.99; H, 5.03; N, 11.66; S, 13.34%. Found: C, 49.79; H, 5.07; N, 11.86; S, 13.53%.

*Ethyl 3-(2-(2-hydroxybenzylidene)hydrazinyl)-3-oxopropanoate* (**8f**). Yield (2.15 g, 86%); m.p. 148–150 °C; IR *ν* 3191–3090 (NH, OH), 1734 (C=O, ester), 1667 (C=O, amide) cm^−1^; ^1^H-NMR *δ* 1.17 (t, *J* = 7.32 Hz, 3H, CH_3_), 3.62 (s, 2H, CH_2_), 4.09 (q, *J* = 7.32 Hz, 2H, CH_2_), 6.82–6.93 (m, 2H, ArH), 7.21–7.31 (m, 1H, ArH), 7.54–7.62 (m, 1H, ArH), 8.27 (s, 1H, –CH=), 9.99 (s, D_2_O exch., 1H, OH), 11.49 (s, D_2_O exch., 1H, NH); ^13^C-NMR *δ* 14.6 (CH_3_), 41.7 (CH_2_), 61.0 (CH_2_), 116.7, 119.9, 120.7, 126.5, 131.7 (ArC), 141.1 (–CH=), 162.1, (ArC), 168.1 (C=O), 168.2 (C=O); MS *m/z*: 251.2 (M^+^+1), 273.1 (M^+^+23). Anal. calcd. for C_12_H_14_N_2_O_4_ (250.25): C, 57.59; H, 5.64; N, 11.19%. Found: C, 57.46; H, 5.67; N, 11.03%.

### 3.4. General Procedure for the Synthesis of N'-Arylidene-2-oxo-2H-chromene-3-carbohydrazides ***6a–f***

To a solution of the appropriate hydrazone **8a**–**f** (1 mmol) and salicaldehyde (**5f**) (0.122 g, 1 mmol) in absolute ethanol (25 mL), a catalytic amount of piperidine (0.3 mL) was added. The reaction mixture was refluxed for 1 h. The formed precipitate was filtered off, washed with ethanol, dried and recrystallized from EtOH/DMF to give hydrazides **6a**–**f**.

*N'-Benzylidene-2-oxo-2H-chromene-3-carbohydrazide* (**6a**). Yield (254 mg, 87%); m.p. 258–260 °C; IR *ν* 3249 (NH), 1700 (C=O, lactone), 1665 (C=O, amide) cm^−1^; ^1^H-NMR *δ* 7.46–7.51 (m, 4H, ArH), 7.55 (d, *J* = 8.1 Hz, 1H, ArH), 7.76–7.77 (m, 3H, ArH), 8.03 (d, *J* = 8.1 Hz, 1H, ArH), 8.48 (s, 1H, –CH=), 8.93 (s, 1H, –CH=), 11.78 (s, D_2_O exch., 1H, NH); ^13^C-NMR *δ* 116.8, 116.9, 118.9, 119.9, 125.8, 127.2, 127.9, 129.4, 130.9, 131.0, 134.5, 134.9, 145.2 (ArC), 148.2 (–CH=), 149.9 (–CH=), 154.5 (C=O); MS *m/z*: 293.1 (M^+^+1), 315.1 (M^+^+23). Anal. calcd. for C_17_H_12_N_2_O_3_ (292.29): C, 69.86; H, 4.14; N, 9.58%. Found: C, 69.58; H, 4.03; N, 9.36%.

*N'-(4-Methoxybenzylidene)-2-oxo-2H-chromene-3-carbohydrazide* (**6b**). Yield (290 mg, 90%); m.p. 265–267 °C; IR *ν* 3257 (NH), 1704 (C=O, lactone), 1666 (C=O, amide) cm^−1^; ^1^H-NMR *δ* 3.82 (s, 3H, OCH_3_), 7.04 (d, *J* = 8.8 Hz, 2H, ArH), 7.48 (t, *J* = 7.4 Hz, 1H, ArH), 7.55 (d, *J* = 8.8 Hz, 1H, ArH), 7.71 (d, *J* = 8.1 Hz, 2H, ArH), 7.79 (t, *J* = 8.1 Hz, 1H, ArH), 7.02 (d, *J* = 7.3 Hz, 1H, ArH), 8.40 (s, 1H, –CH=), 8.90 (s, 1H, –CH=), 11.65 (s, D_2_O exch., 1H, NH); MS *m/z*: 323.2 (M^+^+1). Anal. calcd. for C_18_H_14_N_2_O_4_ (322.31): C, 67.07; H, 4.38; N, 8.69%. Found: C, 66.79; H, 4.44; N, 8.75%.

*N'-(4-Chlorobenzylidene)-2-oxo-2H-chromene-3-carbohydrazide* (**6c**). Yield (265 mg, 81%); m.p. 288–290 °C; IR *ν* 3250 (NH), 1704 (C=O, lactone), 1665 (C=O, amide) cm^−1^; 6.95 (d, *J* = 7.4 Hz, 1H, ArH), 7.48 (t, *J* = 7.3 Hz, 2H, ArH), 7.55 (d, *J* = 8.1 Hz, 2H, ArH), 7.78 (d, *J* = 8.1 Hz, 2H, ArH), 8.02 (d, *J* = 6.6 Hz, 1H, ArH), 8.49 (s, 1H, –CH=), 8.94 (s, 1H, –CH=), 11.95 (s, D_2_O exch., 1H, NH); MS *m/z*: 327.1 (M^+^+1). Anal. calcd. for C_17_H_11_ClN_2_O_3_ (326.73): C, 62.49; H, 3.39; N, 8.57%. Found: C, 62.31; H, 3.39; N, 8.48%.

*N'-(Benzo[d][1,3]dioxol-5-ylmethylene)-2-oxo-2H-chromene-3-carbohydrazide* (**6d**). Yield (259 mg, 77%); m.p. 286–288 °C; IR *ν* 3256 (NH), 1704 (C=O, lactone), 1665 (C=O, amide) cm^−1^; ^1^H-NMR *δ* 6.11 (s, 2H, CH_2_), 6.94–7.79 (m, 6H, ArH), 8.02 (d, *J* = 6.6 Hz, 1H, ArH), 8.38 (s, 1H, –CH=), 8.92 (s, 1H, –CH=), 11.69 (s, D_2_O exch., 1H, NH); MS *m/z*: 337.1 (M^+^+1). Anal. calcd. for C_18_H_12_N_2_O_5_ (336.30): C, 64.29; H, 3.60; N, 8.33%. Found: C, 64.02; H, 3.54; N, 8.52%.

*2-Oxo-N'-(thiophen-2-ylmethylene)-2H-chromene-3-carbohydrazide* (**6e**). Yield (239 mg, 80%); m.p. 282-284 °C; IR *ν* 3291 (NH), 1700 (C=O, lactone), 1664 (C=O, amide) cm^−1^; ^1^H-NMR *δ* 7.45–7.78 (m, 6H, thiophene H, ArH), 8.02 (d, *J* = 8.1 Hz, 1H, ArH), 8.69 (s, 1H, –CH=), 8.91 (s, 1H, –CH=), 11.85 (s, D_2_O exch., 1H, NH); ^13^C-NMR *δ* 116.8 (–CH=), 118.9, 119.9, 125.8, 128.6, 130,1, 130.8, 132.3, 134.9, 139.1 (ArC, thiophene C), 145.1 (–CH=), 148.3, 154.4 (ArC), 158.5 (C=O), 160.4 (C=O); MS *m/z*: 299.1 (M^+^+1). Anal. calcd. for C_15_H_10_N_2_O_3_S (298.32): C, 60.39; H, 3.38; N, 9.39; S, 10.75%. Found: C, 60.36; H, 3.15; N, 9.31; S, 10.59%.

*N'-(2-Hydroxybenzylidene)-2-oxo-2H-chromene-3-carbohydrazide* (**6f**). Yield (271 mg, 88%); m.p. 298–300 °C; IR *ν* 3195–3049 (NH, OH), 1699 (C=O, lactone), 1610 (C=O, amide) cm^−1^; ^1^H-NMR *δ* 6.96 (d, *J* = 6.6 Hz, 2H, ArH), 7.33 (t, *J* = 8.1 Hz, 1H, ArH), 7.48 (t, *J* = 8.1 Hz, 1H, ArH), 7.55-7.57 (m, 3H, ArH, OH), 7.79 (t, *J* = 8.1 Hz, 1H, ArH), 8.02 (d, *J* = 7.3 Hz, 1H, ArH), 0.00 (s, 1H, –CH=), 8.71 (s, 1H, –CH=), 8.92 (s, 1H, –CH=); MS *m/z*: 309.2 (M^+^+1), 331.1 (M^+^+23). Anal. calcd. for C_17_H_12_N_2_O_4_ (308.29): C, 66.23; H, 3.92; N, 9.09%. Found: C, 66.02; H, 4.11; N, 8.92%.

### 3.5. Direct Synthesis of ***6f***

To a solution of hydrazide **7** (0.146 g, 1 mmol) and salicaldehyde (**5f**) (0.244 g, 2 mmol) in absolute ethanol (25 mL), a catalytic amount of piperidine (0.3 mL) was added. The reaction mixture was refluxed for 1 h. The formed precipitate was filtered off, washed with ethanol, dried and recrystallized from EtOH/DMF to give 240 mg of **6f** in 78% yield.

## 4. Conclusions

We have identified another main product, confirmed as malohydrazide (**4**), besides salicylaldehyde azine (**3**) which was reported by Soliman *et al.* in 1985 [38] as the sole product of the title reaction. Whereas the observations of this investigation have confirmed the identity of salicylaldehyde azine (**3**) and malonohydrazide (**4**), as the compounds produced through the reaction of ester **1** with hydrazine hydrate, the structures of products claimed to be formed from hydrazide **2** in investigations reported over the last two decades [25,26,27,28,29,30,31,32,33,34,35,36] need to be reassigned. Moreover, we described an efficient synthetic route for our targeted hydrazones **6a**–**f**. Generalization of the latter established method can be widely used in synthesis of library of coumarin-based hydrazones.
